# An improved sliding mode approach for trajectory following control of nonholonomic mobile AGV

**DOI:** 10.1038/s41598-022-22697-w

**Published:** 2022-10-22

**Authors:** Benchi Jiang, Jiankang Li, Siyang Yang

**Affiliations:** School of Mechanical Engineering, Anhui Polytechnic University, Wuhu, 241000 Anhui People’s Republic of China

**Keywords:** Mechanical engineering, Electrical and electronic engineering

## Abstract

This paper attempts to address the trajectory following control problem of nonholonomic mobile AGV by proposing an improved sliding mode control approach in which, based on the kinematics and attitude deviations established for AGV, the motion characteristics are analyzed and a backstepping sliding mode control with a novel reaching law is designed. This reaching law integrates the merits of the power and exponential reaching laws and promotes the convergence rates of tracking errors. Moreover, with the improved sliding mode controller, the asymptotic stability of tracking deviations can be strictly guaranteed. The simulations have demonstrated the effectiveness and superiority of the proposed approach for mobile AGV.

## Introduction

AGVs are widely used in scientific studies, transportation, and research because of their high degrees of automation, flexibility, and anti-interference capability. As one of the core technologies of AGVs, trajectory tracking control has attracted much research attention, and various control methods have been proposed for this purpose, including robust control, backstepping control, PID control, fuzzy control, adaptive control, and sliding mode control^1–6^. Sliding mode control, as an effective method for nonlinear systems, has the advantages of fast response and insensitivity to parameter changes and external disturbances. However, this method is also prone to jitter, slow convergence, and low tracking accuracy, thereby reducing the stability of the system.

Many studies have been conducted to effectively utilize the advantages of sliding mode control and alleviate its adverse effects on the control system. For instance, Sun et al.^7^ presented an adaptive integral terminal sliding mode (AITSM) control algorithm for a trajectory-tracking task that exhibits great superiority in tracking precision and control robustness. Hamid et al.^8^ combined the optimal and robust control system with adaptive gains to follow the desired path. By comparing the results of this approach with those of an adaptive sliding mode control (ASMC), they found that their proposed controller exerts less control effort. To improve the accuracy of trajectory tracking control, an enhanced variable structure based on sliding mode has also been designed. The projected trajectory track control technique can improve the power of mobile robots and minimize the error of a pose^9^. A continuous sliding mode control (CSMC) scheme is also developed for high-precision trajectory tracking tasks. This approach not only avoids chattering but also guarantees the stability of the system^10^. A fractional-order sliding mode fault-tolerant control method is proposed to follow the desired path. This method can rapidly converge all error states to zero, and generates minimal tracking error chattering^11^. Ameni et al.^12^ proposed a sliding mode controller with adaptive gains for trajectory tracking, thereby ensuring accuracy and minimizing the tracking errors and chattering effects. A novel trajectory tracking control method has also been proposed for nonholonomic mobile robots based on the non-negative piecewise predefined-time theorem. The target method rapidly converts the error to zero in a certain period^13^. A new integral high-order sliding mode (IHOSM) surface is also introduced to achieve a rapid and accurate trajectory tracking^14^. The sliding mode control method based on the improved reaching law is developed to make the system arrive at the sliding surface rapidly and effectively follow the different trajectories^15^. Xie et al.^16^ studied a new coupled fractional-order sliding mode control (CFSMC) with superior capacities for providing additional control flexibility and achieving high accuracy.

Although the references mentioned above have made contributed to the application of sliding mode control to trajectory tracking, the problem of difficult control resulting from the complex nonlinear system of the AGV itself remains unaddressed. In building the kinematic model of AGV, a sliding mode trajectory tracking controller with an improved reaching law is designed to further improve the convergence speed and tracking accuracy. The stability of the tracking control law is discussed using Lyapunov’s stability theorem, and the global asymptotic stability of the system is validated. Simulation results show that the proposed controller has better dynamic quality than other sliding mode controllers, thereby improving the convergence speed and tracking accuracy of the system and effectively suppressing system jitter.

The rest of this paper is organized as follows. “Dynamical modeling for mobile AGV” section presents the kinematics of mobile AGV. “Reaching law design” section shows the improved reaching law and stability proof. “Improved sliding mode control design” section presents the enhanced sliding mode control design. “Numerical illustrations” section analyzes the simulation results and compares the proposed method with other schemes. “Conclusion” section draws the conclusions.

## Dynamical modeling for mobile AGV

**Figure 1:**
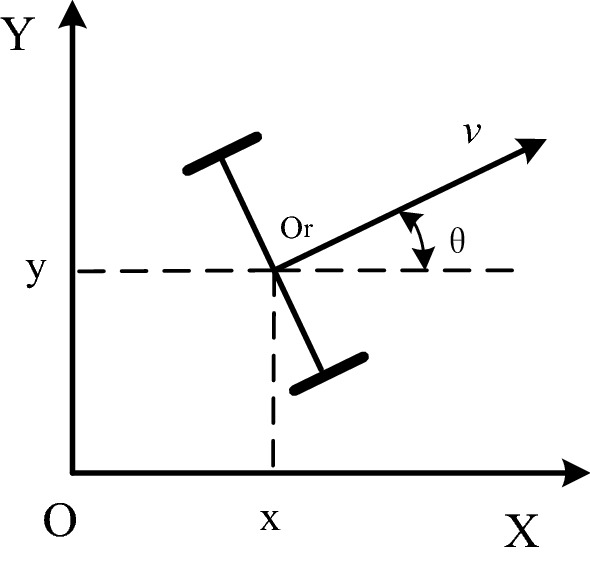
Schematic of mobile AGV.

The schematic of the mobile AGV is shown in Fig. 1. The structure consists of two actuated wheels and two passive wheels, and the front wheels mainly support the body of the mobile AGV, whereas the rear wheels drive the vehicle. The steering of mobile AGV can be achieved by regulating the speed difference between two actuated wheels.

From Fig. 1, the kinematic model of mobile AGV in the global coordinate system XOY can be derived as

1 $$\left[ {\begin{array}{*{20}c} {\dot{x}} \\ {\dot{y}} \\ {\dot{\theta }} \\ \end{array} } \right] = \left[ {\begin{array}{*{20}c} {\begin{array}{*{20}c} {\cos \theta } \\ {\sin \theta } \\ 0 \\ \end{array} } & {\begin{array}{*{20}c} 0 \\ 0 \\ 1 \\ \end{array} } \\ \end{array} } \right] \cdot \left[ {\begin{array}{*{20}c} v \\ \omega \\ \end{array} } \right],$$

where $v$ denotes the linear velocity of point $O_{r}$, and $\omega$ represents the angular velocity. Let the counterclockwise rotation of AGV be the positive direction. Given that two actuated wheels control the translational movement and process of mobile AGV, the linear and angular velocities of mobile AGV can be determined as

2 $$\left\{ {\begin{array}{*{20}l} {v = \left( {v_{l} + v_{r} } \right)/2} \hfill \\ {\omega = \left( {v_{l} - v_{r} } \right)/\left( {2L} \right)} \hfill \\ \end{array} } \right.,$$

or more directly,

3 $$\left[ {\begin{array}{*{20}c} v \\ \omega \\ \end{array} } \right] = \frac{1}{2}\left[ {\begin{array}{*{20}c} 1 & 1 \\ \frac{1}{L} & { - \frac{1}{L}} \\ \end{array} } \right] \cdot \left[ {\begin{array}{*{20}c} {v_{l} } \\ {v_{r} } \\ \end{array} } \right],$$

where $v_{l}$ and $v_{r}$ are the linear velocities of the left and right actuated wheels, respectively, and $L$ denotes the distance from point $O_{r}$ to each actuated wheel. In the global coordinate system, by the expected position $p_{r} = \left[ {x_{r} ,y_{r} ,\theta_{r} } \right]^{{\text{T}}}$ and actual position $p = \left[ {x,y,\theta } \right]^{{\text{T}}}$, the tracking error model of mobile AGV can be derived as

4 $$e = \left[ {\begin{array}{*{20}c} {x_{e} } \\ {y_{e} } \\ {\theta_{e} } \\ \end{array} } \right] = \left[ {\begin{array}{*{20}c} {\cos \theta } & {\sin \theta } & 0 \\ { - \sin \theta } & {\cos \theta } & 0 \\ 0 & 0 & 1 \\ \end{array} } \right] \cdot \left[ {\begin{array}{*{20}c} {x_{r} - x} \\ {y_{r} - y} \\ {\theta_{r} - \theta } \\ \end{array} } \right],$$

where $x_{e}$, $y_{e}$, and $\theta_{e}$ represent the tracking errors of the X-axis, Y-axis, and yaw attitude, respectively. Substituting Eq. (1) into the differential version of Eq. (4) yields

5 $$\dot{e} = \left[ {\begin{array}{*{20}c} {\dot{x}_{e} } \\ {\dot{y}_{e} } \\ {\dot{\theta }_{e} } \\ \end{array} } \right] = \left[ {\begin{array}{*{20}c} {y_{e} \omega + v_{r} \cos \theta_{e} - v} \\ {v_{r} \sin \theta_{e} - x_{e} \omega } \\ {\omega_{r} - \omega } \\ \end{array} } \right],$$

where $v_{r}$ and $\omega_{r}$ denote the desired linear and angular velocities of mobile AGV, respectively. On the basis of the error system in Eq. (5), the control goal of this research is to drive the actual position of mobile AGV toward its expected path $p_{r} = \left[ {x_{r} ,y_{r} ,\theta_{r} } \right]^{{\text{T}}}$ by designing the control input $q = \left[ {v,\omega } \right]^{{\text{T}}}$, that is, to ensure that the tracking error $e = \left[ {x_{e} ,y_{e} ,\theta_{e} } \right]^{{\text{T}}}$ is bounded and $\mathop {\lim }\limits_{t \to \infty } \left\| {\left[ {x_{e} ,y_{e} ,\theta_{e} } \right]} \right\|^{{\text{T}}} = 0$.

**Figure 2:**
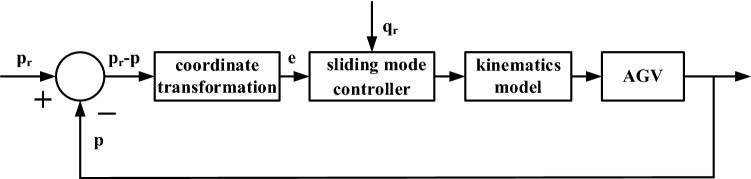
Trajectory tracking control structure block diagram.

By combining the abovementioned equations with the AGV kinematic model, the trajectory tracking control structure block diagram can be built as shown in Fig. 2. Under this system block diagram, the positional error of the system can be brought to 0, hence enabling the AGV to track the desired trajectory smoothly.

## Reaching law design

The kinematic model of AGV is a multi-input nonlinear complex system. Due to research contributions over the years, sliding mode control can be smoothly implemented in such a system. The backstepping method is then employed to design the sliding mode control switching function^17^.

### Lemma 1

For $\forall x \in {\mathbb{R}}$ and $\left| x \right| < \infty$, $\varphi \left( x \right) = x\sin \left( {{\text{arc}}\tan x} \right) \ge 0$, besides, $\varphi \left( x \right) = 0$ if and only if $x = 0$^18^.

### Proof

If $x = 0$, then $\varphi \left( x \right) = 0$; while if $x \in \left( {0, + \infty } \right)$, then ${\text{arc}}\tan x \in \left( {0,\pi /2} \right)$. Therefore, $\sin \left( {{\text{arc}}\tan x} \right) > 0$, $\varphi \left( x \right) > 0$; otherwise, for $x \in \left( { - \infty ,0} \right)$, ${\text{arc}}\tan x \in \left( { - \pi /2,0} \right)$. Therefore, $\sin \left( {{\text{arc}}\tan x} \right) < 0$, $\varphi \left( x \right) > 0$.

Let $x_{e} = 0$, $V_{y} = 0.5y_{e}^{2}$ and $\theta_{e} = - {\text{arc}}\tan \left( {v_{r} y_{e} } \right)$, then $\dot{V}_{y}$ turns into

6 $$\begin{aligned} \dot{V}_{y} = & y_{e} \cdot \dot{y}_{e} = y_{e} \left( {v_{r} \sin \theta_{e} - x_{e} \omega } \right) \\ = & - y_{e} v_{r} \sin \left( {{\text{arc}}\tan \left( {v_{r} y_{e} } \right)} \right) - x_{e} y_{e} \omega . \\ \end{aligned}$$

Subject to Lemma 1,

7 $$\dot{V}_{y} \le 0,$$

if and only if $v_{r} y_{e} = 0$.

From above, if $x_{e} = 0$ and $\theta_{e} = - {\text{arc}}\tan \left( {v_{r} y_{e} } \right)$, then the tracking error of each orientation converges to 0. As a result, the sliding function $s$ is given as

8 $$s = \left[ {\begin{array}{*{20}c} {s_{1} } \\ {s_{2} } \\ \end{array} } \right] = \left[ {\begin{array}{*{20}c} {x_{e} } \\ {\theta_{e} + {\text{arc}}\tan \left( {v_{r} y_{e} } \right)} \\ \end{array} } \right].$$

Therefore, by designing the sliding mode controller to keep $s_{1}$ and $s_{2}$ tending to 0, $x_{e}$ tends to 0, whereas $\theta_{e}$ tends to $- {\text{arc}}\tan \left( {v_{r} y_{e} } \right)$; eventually, $y_{e}$ and $\theta_{e}$ both tend to 0.

The sliding mode motion is divided into two processes, namely, convergence motion and sliding mode motion. Convergence motion is the process by which $s$ tends to 0. According to the principle of sliding mode control, the sliding mode reachability condition only indicates that the system motion point reaches the switching surface in finite time at any position in space but not in what way. In this regard, the motion quality of the concurrent motion can be enhanced by the reaching law.

To remedy the drawbacks of conventional reaching laws, such as $\dot{s} = - \,{\epsilon}{\text{sgn}} s$, $\dot{s} = - \,{\epsilon}{\text{sgn}} s - ks$ and $\dot{s} = - \,{\epsilon}{\text{sgn}} s - f\left( s \right)$ where $\,{\epsilon} > 0$, $k > 0$, the following power reaching law is employed to construct the expected sliding mode controller:

9 $$\dot{s} = - \,{\epsilon}\left| s \right|^{\alpha } {\text{sgn}} s.$$

Equation (9) manifests that by properly regulating the parameter $\alpha$, higher convergence rates can be achieved if the large initial states stay far away from the sliding surfaces. By contrast, when the system states approach the sliding surfaces, the chattering of tracking control can be well reduced^19,20^.

Considering the merits of the power reaching law, the following double power reaching law has been recently proposed for mobile AGV ^15^:

10 $$\dot{s} = - \,{\epsilon}_{1} |s|^{\alpha } {\text{sgn}} s - \,{\epsilon}_{2} |s|^{\beta } {\text{sgn}} s,$$

where $\,{\epsilon}_{1,2} > 0$, $0 < \alpha < 1$, and $\beta > 1$. The convergence motion is divided into two phases by $s = 1$ in Eq. (10). If $\left| s \right| < 1$, then the first phase plays a significant role. If $\left| s \right| > 1$, then the second stage plays an important role.

Segmentation control leads to a discontinuity when the system reaches a boundary. By adding an exponential term, the segmental control can be adjusted. The exponential time accelerates the convergence of the intermediate state of the double power reaching law. The system arrives at the switching surface as an asymptotic process for the exponential reaching law but not in a certain period. An equal velocity convergence term is required to ensure the existence of the sliding mode. When $s$ tends to 0, the convergence velocity is $\,{\epsilon}$ instead of 0. Therefore, the system is guaranteed to arrive within a specific time. The reaching law can be designed as follows:

11 $$\dot{s} = - \,{\epsilon}_{1} |s|^{\alpha } \, {\text{sgn}} {{s}} - \,{\epsilon}_{2} |s|^{\beta } \, {\text{sgn}} {\text{s}} - \,{\epsilon}_{3} {\text{sgn}} s - ks,$$

where $$k > 0$$ and $$\,{\epsilon}_{3} > 0$$.

In a situation where AGV is subject to bounded disturbances in actual operation, the following theorem is used to prove the stability of the system:

### Theorem 1

When AGV is subject to bounded disturbances during operation, the system meets the following conditions of global stability:

12 $$\left| {s_{1} } \right| \le \min \left\{ {\left[ {\frac{{d_{1} - \,{\epsilon}_{13} }}{{\,{\epsilon}_{11} }}} \right]^{{\frac{1}{{\alpha_{1} }}}} ,\left[ {\frac{{d_{1} - \,{\epsilon}_{13} }}{{\,{\epsilon}_{12} }}} \right]^{{\frac{1}{{\beta_{1} }}}} } \right\},d_{1} \ge \,{\epsilon}_{13} ,$$

13 $$\left| {s_{2} } \right|\,{\le}\min \left\{ {\left[ {\frac{{d_{2} - \,{\epsilon}_{23} }}{{\,{\epsilon}_{21} }}} \right]^{{\frac{1}{{\alpha_{2} }}}} ,\left[ {\frac{{d_{2} - \,{\epsilon}_{23} }}{{\,{\epsilon}_{22} }}} \right]^{{\frac{1}{{\beta_{2} }}}} } \right\},d_{2} \ge \,{\epsilon}_{23} .$$

### Proof

By leading the bounded disturbance terms *D*_1_ and *D*_2_ into the improved reaching law, then

14 $$\dot{s} = \left[ {\begin{array}{*{20}l} {D_{1} - \,{\epsilon}_{11} \left| {s_{1} } \right|^{{\alpha_{1} }} {\text{sgn}} s - \,{\epsilon}_{12} \left| {s_{1} } \right|^{{\beta_{1} }} {\text{sgn}} s - k_{1} s_{1} - \,{\epsilon}_{13} {\text{sgn}} s} \hfill \\ {D_{2} - \,{\epsilon}_{21} \left| {s_{2} } \right|^{{\alpha_{2} }} {\text{sgn}} s - \,{\epsilon}_{22} \left| {s_{2} } \right|^{{\beta_{2} }} {\text{sgn}} s - k_{2} s_{2} - \,{\epsilon}_{23} {\text{sgn}} s} \hfill \\ \end{array} } \right],$$

where $$D_{1} \le d_{1}$$ and $$D_{2} \le d_{2}$$.

By selecting the Lyapunov function $V = 0.5s^{2}$, then

15 $$\begin{aligned} \dot{V} = & - \,{\epsilon}_{11} \left| {s_{1} } \right|^{{\alpha_{1} + 1}} - \,{\epsilon}_{21} \left| {s_{2} } \right|^{{\alpha_{2} + 1}} - \,{\epsilon}_{12} \left| {s_{1} } \right|^{{\beta_{1} + 1}} - \,{\epsilon}_{22} \left| {s_{2} } \right|^{{\beta_{2} + 1}} - k_{1} s_{1}^{2} - k_{2} s_{2}^{2} - \,{\epsilon}_{13} \left| {s_{1} } \right| - \,{\epsilon}_{23} \left| {s_{2} } \right| + \left| {s_{1} } \right|\left| {D_{1} } \right| + \left| {s_{2} } \right|\left| {D_{2} } \right|\, \, \\ = & - \,{\epsilon}_{12} \left| {s_{1} } \right|^{{\beta_{1} + 1}} - \,{\epsilon}_{22} \left| {s_{2} } \right|^{{\beta_{2} + 1}} - k_{1} s_{1}^{2} - k_{2} s_{2}^{2} - \left| {s_{1} } \right|\left( {\,{\epsilon}_{13} + \,{\epsilon}_{11} \left| {s_{1} } \right|^{{\alpha_{1} }} - \left| {D_{1} } \right|} \right) - \left| {s_{2} } \right|\left( {\,{\epsilon}_{23} + \,{\epsilon}_{21} \left| {s_{2} } \right|^{{\alpha_{2} }} - \left| {D_{2} } \right|} \right), \\ \end{aligned}$$

16 $$\begin{aligned} \dot{V} = & - \,{\epsilon}_{11} \left| {s_{1} } \right|^{{\alpha_{1} + 1}} - \,{\epsilon}_{21} \left| {s_{2} } \right|^{{\alpha_{2} + 1}} - \,{\epsilon}_{12} \left| {s_{1} } \right|^{{\beta_{1} + 1}} - \,{\epsilon}_{22} \left| {s_{2} } \right|^{{\beta_{2} + 1}} - k_{1} s_{1}^{2} - k_{2} s_{2}^{2} - \,{\epsilon}_{13} \left| {s_{1} } \right| - \,{\epsilon}_{23} \left| {s_{2} } \right| + \left| {s_{1} } \right|\left| {D_{1} } \right| + \left| {s_{2} } \right|\left| {D_{2} } \right|\, \, \\ = & - \,{\epsilon}_{12} \left| {s_{1} } \right|^{{\beta_{1} + 1}} - \,{\epsilon}_{22} \left| {s_{2} } \right|^{{\beta_{2} + 1}} - k_{1} s_{1}^{2} - k_{2} s_{2}^{2} - \left| {s_{1} } \right|\left( {\,{\epsilon}_{13} + \,{\epsilon}_{11} \left| {s_{1} } \right|^{{\alpha_{1} }} - \left| {D_{1} } \right|} \right) - \left| {s_{2} } \right|\left( {\,{\epsilon}_{23} + \,{\epsilon}_{21} \left| {s_{2} } \right|^{{\alpha_{2} }} - \left| {D_{2} } \right|} \right). \\ \end{aligned}$$

From Eqs. (12) and (13),

17 $$\,{\epsilon}_{13} + \,{\epsilon}_{11} \left| {s_{1} } \right|^{{\alpha_{1} }} \ge \left| {D_{1} } \right|,\,{\epsilon}_{23} + \,{\epsilon}_{21} \left| {s_{2} } \right|^{{\alpha_{2} }} \ge \left| {D_{2} } \right|.$$

Therefore,

18 $$\dot{V} \le 0.$$

In this case, if $\left| {s_{1} } \right| \le \left[ {\frac{{d_{1} - \,{\epsilon}_{13} }}{{\,{\epsilon}_{11} }}} \right]^{{\frac{1}{{\alpha_{1} }}}}$, $\left| {s_{2} } \right| \le \left[ {\frac{{d_{2} - \,{\epsilon}_{23} }}{{\,{\epsilon}_{21} }}} \right]^{{\frac{1}{{\alpha_{2} }}}}$, then the AGV tracking control system can reach convergence in a limited time.

Similarly, the above equation can be transformed as

19 $$\begin{aligned} \dot{V} = & - \,{\epsilon}_{11} \left| {s_{1} } \right|^{{\alpha_{1} + 1}} - \,{\epsilon}_{21} \left| {s_{2} } \right|^{{\alpha_{2} + 1}} - \,{\epsilon}_{12} \left| {s_{1} } \right|^{{\beta_{1} + 1}} - \,{\epsilon}_{22} \left| {s_{2} } \right|^{{\beta_{2} + 1}} - k_{1} s_{1}^{2} - k_{2} s_{2}^{2} - \,{\epsilon}_{13} \left| {s_{1} } \right| - \,{\epsilon}_{23} \left| {s_{2} } \right| + \left| {s_{1} } \right|\left| {D_{1} } \right| + \left| {s_{2} } \right|\left| {D_{2} } \right|\, \, \\ = & - \,{\epsilon}_{11} \left| {s_{1} } \right|^{{\alpha_{1} + 1}} - \,{\epsilon}_{21} \left| {s_{2} } \right|^{{\alpha_{2} + 1}} - k_{1} s_{1}^{2} - k_{2} s_{2}^{2} - \left| {s_{1} } \right|\left( {\,{\epsilon}_{13} + \,{\epsilon}_{12} \left| {s_{1} } \right|^{{\beta_{1} }} - \left| {D_{1} } \right|} \right) - \left| {s_{2} } \right|\left( {\,{\epsilon}_{23} + \,{\epsilon}_{22} \left| {s_{2} } \right|^{{\beta_{2} }} - \left| {D_{2} } \right|} \right). \\ \end{aligned}$$

Therefore, $$\left| {s_{1} } \right| \le \left[ {\frac{{d_{1} - \,{\epsilon}_{13} }}{{\,{\epsilon}_{12} }}} \right]^{{\frac{1}{{\beta_{1} }}}}$$, $$\left| {s_{2} } \right| \le \left[ {\frac{{d_{2} - \,{\epsilon}_{23} }}{{\,{\epsilon}_{22} }}} \right]^{{\frac{1}{{\beta_{2} }}}}$$.

In sum, the controller can exhibit better robustness in the presence of external disturbances.

**Figure 3:**
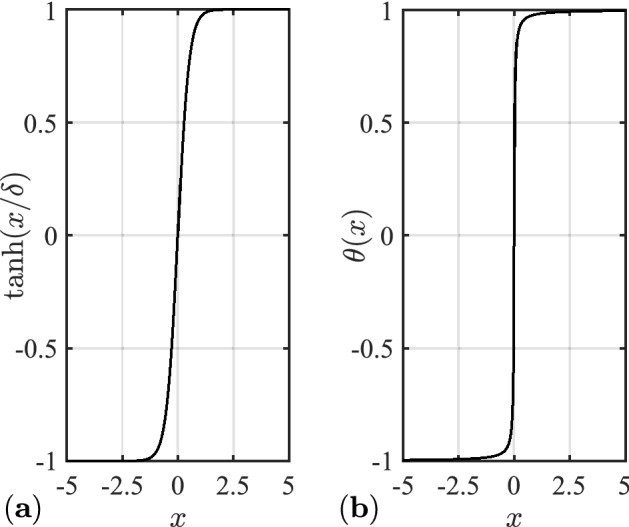
(**a**) Hyperbolic tangent function; (**b**) continuous function.

To further reduce the chattering of sliding mode control, the hyperbolic tangent function

20 $$\tanh \left( {\frac{x}{\sigma }} \right) = \frac{{e^{{\frac{x}{\sigma }}} - e^{{ - \frac{x}{\sigma }}} }}{{e^{{\frac{x}{\sigma }}} + e^{{ - \frac{x}{\sigma }}} }},$$

and continuous function

21 $$\theta (s) = \frac{s}{|s| + \delta },$$

are applied to replace the sign function, where $\sigma ,\delta > 0$. The hyperbolic tangent function when $\sigma = 0.5$ and the continuous function when $\delta = 0.02$ are shown in Fig. 3.

The improved reaching law is given as

22 $$\dot{s} = - \,{\epsilon}_{1} |s|^{\alpha } \frac{s}{|s| + \delta } - \,{\epsilon}_{2} |s|^{\beta } \frac{s}{|s| + \delta } - ks - \,{\epsilon}_{3} \tanh \left( {\frac{s}{\sigma }} \right).$$

The stability of the improved reaching law is then demonstrated in Eq. (21) by the Lyapunov function.

By selecting the Lyapunov function $V = 0.5s^{2}$,

23 $$\dot{V} = s\dot{s} = - \,{\epsilon}_{1} \left| s \right|^{\alpha } \frac{{s^{2} }}{\left| s \right| + \delta } - \,{\epsilon}_{2} \left| s \right|^{\beta } \frac{{s^{2} }}{\left| s \right| + \delta } - ks^{2} - \,{\epsilon}_{3} s\tanh \left( {\frac{s}{\sigma }} \right).$$

Given that the first three terms in Eq. (22) are negative definite, only the last term, $\,{\epsilon}_{3} s\tanh \left( {s/\sigma } \right)$, needs to be analyzed.

In the light of the lemma offered in Ref.^21^, for $\forall x$ and $\exists \sigma > 0$,

24 $$x\tanh \left( {\frac{x}{\sigma }} \right) = \left| {x\tanh \left( {\frac{x}{\sigma }} \right)} \right| = \left| x \right|\left| {\tanh \left( {\frac{x}{\sigma }} \right)} \right| \ge 0,$$

then,

25 $$\,{\epsilon}_{3} s\tanh \left( {s/\sigma } \right) \ge 0.$$

Therefore,

26 $$\dot{V} \le 0.$$

From the above proof, the improved reaching law allows AGV to reach the sliding mode surface from its initial position.

## Improved sliding mode control design

With the proposed smooth functions and power reaching law, the sliding mode control design is given as

27 $$\left[ {\begin{array}{*{20}c} v \\ \omega \\ \end{array} } \right] = \left[ {\begin{array}{*{20}c} {y_{e} \omega + v_{r} \cos \theta_{e} - \dot{s}_{1} } \\ {\frac{{\omega_{r} + \frac{\partial \rho }{{\partial v_{r} }}\dot{v}_{r} + \frac{\partial \rho }{{\partial y_{e} }}\left( {v_{r} \sin \theta_{e} } \right) - \dot{s}_{2} }}{{1 + \frac{\partial \rho }{{\partial y_{e} }}x_{e} }}} \\ \end{array} } \right]$$

where $\rho = {\text{arc}}\tan \left( {v_{r} y_{e} } \right)$, $\frac{\partial \rho }{{\partial v_{r} }} = \frac{{y_{e} }}{{1 + \left( {v_{r} y_{e} } \right)^{2} }}$, and $\frac{\partial \rho }{{\partial y_{e} }} = \frac{{v_{r} }}{{1 + \left( {v_{r} y_{e} } \right)^{2} }}$. The derivation of Eq. (26) is then given as follows.

By differentiating Eq. (8) concerning time,

28 $$\dot{s} = \left[ {\begin{array}{*{20}c} {\dot{s}_{1} } \\ {\dot{s}_{2} } \\ \end{array} } \right] = \left[ {\begin{array}{*{20}c} {\dot{x}_{e} } \\ {\dot{\theta }_{e} + \frac{\partial \rho }{{\partial v_{r} }}\dot{v}_{r} + \frac{\partial \rho }{{\partial y_{e} }}\dot{y}_{e} } \\ \end{array} } \right].$$

Substituting Eq. (5) into Eq. (27) yields

29 $$\dot{s} = \left[ {\begin{array}{*{20}c} {y_{e} \omega + v_{r} \cos \theta_{e} - v} \\ {\omega_{r} - \omega + \frac{\partial \rho }{{\partial v_{r} }}\dot{v}_{r} + \frac{\partial \rho }{{\partial y_{e} }}\left( {v_{r} \sin \theta_{e} - x_{e} \omega } \right)} \\ \end{array} } \right].$$

By using the improved reaching law in Eq. (21),

30 $$\dot{s} = \left[ {\begin{array}{*{20}c} {\dot{s}_{1} } \\ {\dot{s}_{2} } \\ \end{array} } \right] = \left[ {\begin{array}{*{20}l} { - \,{\epsilon}_{11} \left| {s_{1} } \right|^{{\alpha_{1} }} \frac{{s_{1} }}{{\left| {s_{1} } \right| + \delta_{1} }} - \,{\epsilon}_{12} \left| {s_{1} } \right|^{{\beta_{1} }} \frac{{s_{1} }}{{\left| {s_{1} } \right| + \delta_{1} }} - k_{1} s_{1} - \,{\epsilon}_{13} \tanh \left( {\frac{{s_{1} }}{{\sigma_{1} }}} \right)} \hfill \\ { - \,{\epsilon}_{21} \left| {s_{2} } \right|^{{\alpha_{2} }} \frac{{s_{2} }}{{\left| {s_{2} } \right| + \delta_{2} }} - \,{\epsilon}_{22} \left| {s_{2} } \right|^{{\beta_{2} }} \frac{{s_{2} }}{{\left| {s_{2} } \right| + \delta_{2} }} - k_{2} s_{2} - \,{\epsilon}_{23} \tanh \left( {\frac{{s_{2} }}{{\sigma_{2} }}} \right)} \hfill \\ \end{array} } \right].$$

From Eqs. (28) and (29), the result in Eq. (26) can be easily deduced. By using the designed sliding mode controller, the tracking errors of mobile AGV become asymptotically stable.

## Numerical illustrations

To sufficiently verify the effectiveness and superiority of the proposed control scheme, the controller presented in Ref.^22^ is selected for a comparison. The control parameters used in the simulations are as follows:

For the desired linear path, $v_{r} = 1m{/}s$, $\omega_{r} = 0rad{/}s$, $x_{r} = t$, $y_{r} = t$, $\theta_{r} = \pi /4$, $p_{r} = \left[ {0,0, \pi {/}4} \right]$, and $p = \left[ { - 2, - 3, - \pi {/}4} \right]$.

For the desired circular path, $v_{r} = 1\,{\text{m/s}}$, $\omega_{r} = 1\,{\text{rad/s}}$, $x_{r} = \cos t$, $y_{r} = \sin t$, $\theta_{r} = t$, $p_{r} = \left[ {1,0,{{\pi /2}}} \right]$, and $p = \left[ { - 2, - 0.5,{{\pi /4}}} \right]$.

For the several critical parameters, $\,{\epsilon}_{11} = \,{\epsilon}_{21} = 6$, $\alpha_{1} = \alpha_{2} = 0.6$, $\delta_{1} = \delta_{2} = 0.02$, $\,{\epsilon}_{12} = \,{\epsilon}_{22} = 6$, $\beta_{1} = \beta_{2} = 8$, $k_{1} = k_{2} = 20$, $\,{\epsilon}_{13} = \,{\epsilon}_{23} = 10$, and $\sigma_{1} = \sigma_{2} = 0.5$. The running time is set to 20 s, whereas the sampling time is 0.1 s.

Given that the AGV trajectory tracking control system is subject to external disturbances during operation, the disturbance $D(t) = \sin (t)$ is chosen to verify the robustness of the control system.

**Figure 4:**
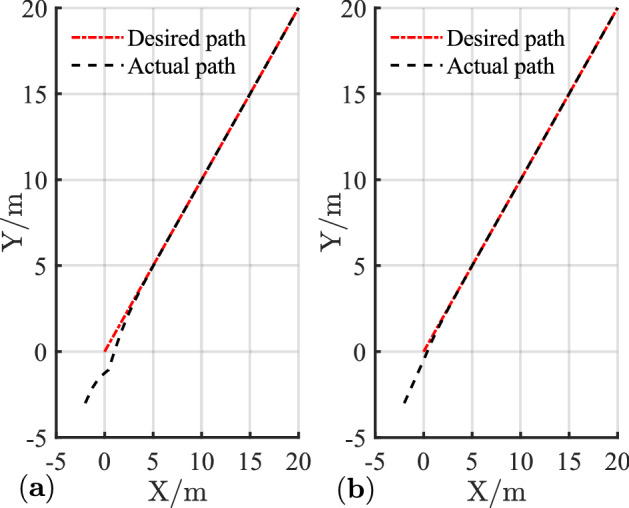
(**a**) Tracking controller in Ref.[^22^]; (**b**) proposed control.

**Figure 5:**
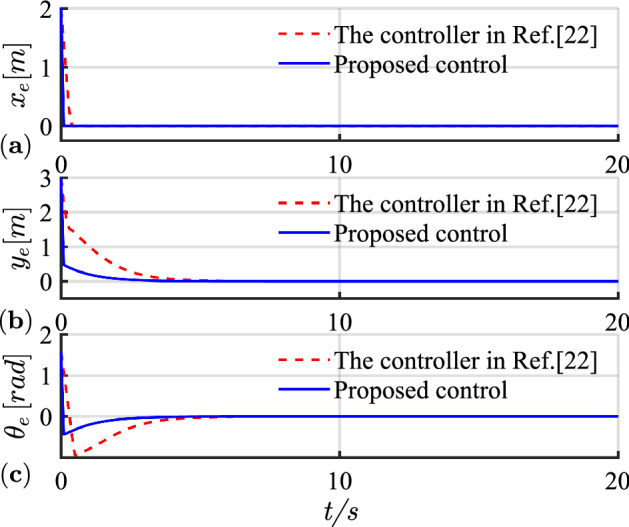
(**a**) Tracking error of X-axis; (**b**) tracking error of Y-axis; (**c**) tracking error of yaw attitude.

**Figure 6:**
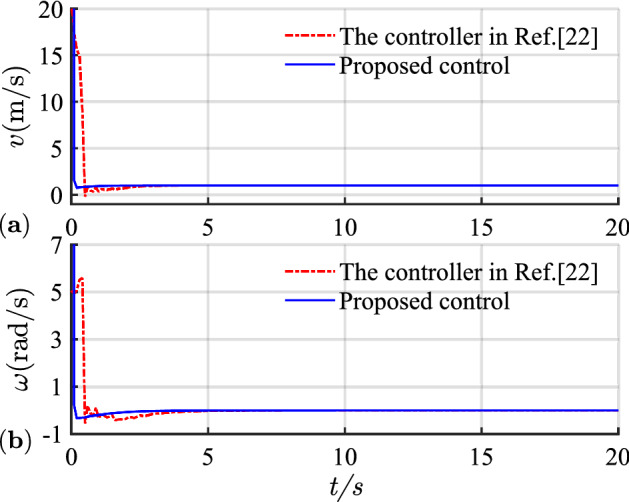
(**a**) Linear velocity; (**b**) angular velocity.

**Figure 7:**
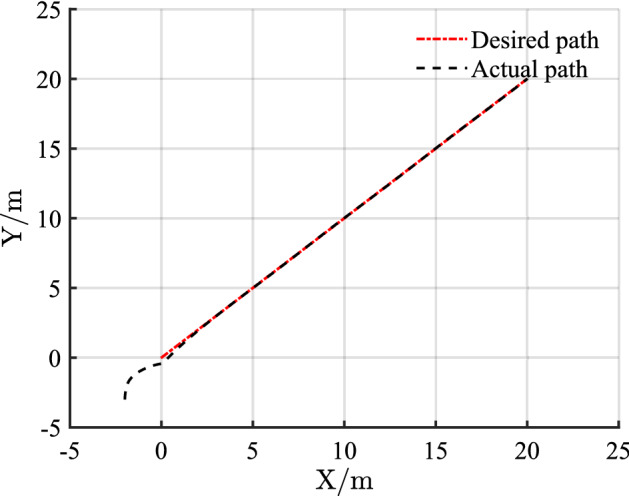
Linear trajectory tracking with disturbance.

**Figure 8:**
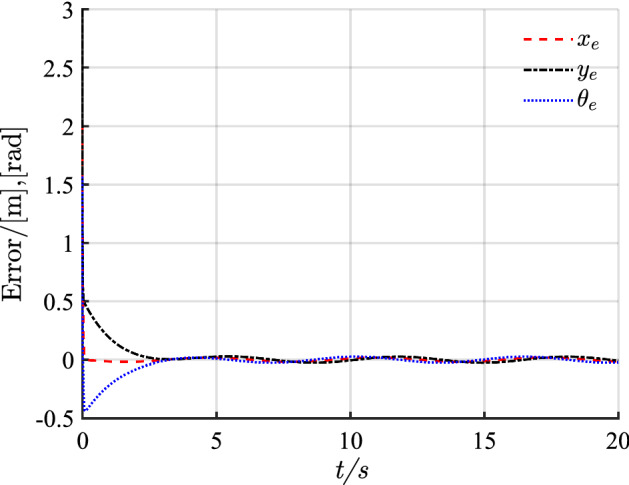
Pose error with disturbance.

**Figure 9:**
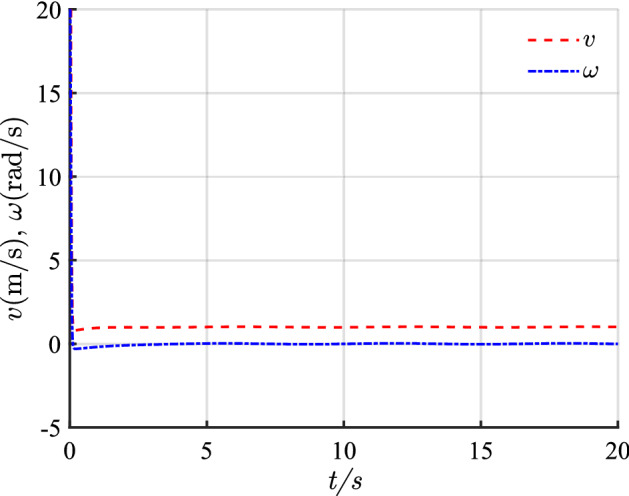
Control input with disturbance.

The simulation results of linear trajectory tracking for comparison with Ref.^22^ are shown in Figs. 4, 5 and 6, whereas the simulation results of linear trajectory tracking for comparison with the proposed controller adding disturbance $D(t)$ are shown in Figs. 7, 8 and 9.

Relative to the scheme proposed in Ref. ^22^, the controller proposed in this study demonstrates faster and smoother performance in following the desired linear path under the same positional error condition in Fig. 4. Figure 5 shows the X-axis, Y-axis tracking errors, and yaw attitude. For the initial position error, the proposed controller rapidly and steadily converts the error to zero, hence reflecting the merits of the exponential term in the reaching law, which can improve the speed of error convergence at more significant errors. From Fig. 6, the linear and angular velocities of the proposed controller converge near the desired value in a brief period and then connect flatly to the desired value. With the hyperbolic tangent and continuous function, the jitter of the system is reduced substantially.

Figure 7 shows that with the disturbance $D(t)$, the desired path is tracked quickly and accurately after a slight fluctuation, and the tracking results are almost unaffected. As shown in Fig. 8, the positional error also shows slight fluctuations after the disturbance. These fluctuations only have a slight effect on the control system. Given the good anti-interference properties of the sliding mode control and the excellent controller design, the control input is hardly affected as shown in Fig. 9.

Combining Figs. 4, 5, 6, 7, 8 and 9 shows that the proposed controller not only improves the convergence speed and anti-interference ability during linear trajectory tracking but also eliminates the jitter phenomenon of the system itself. The simulation results verify the effectiveness of the provided control strategy, which improves the tracking effect and motion quality of the AGV in trajectory tracking.

**Figure 10:**
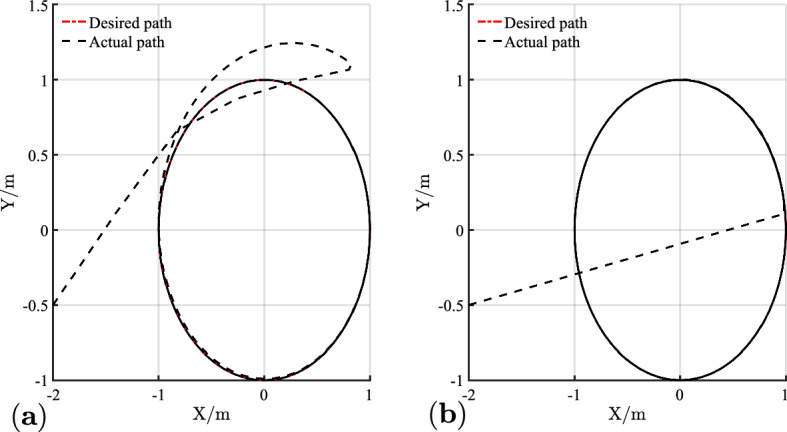
(**a**) Tracking controller in Ref.[^22^]; (**b**) proposed control.

**Figure 11:**
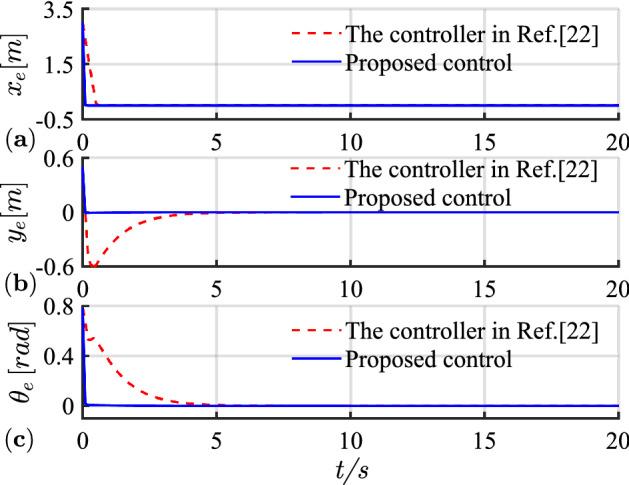
(**a**) Tracking error of X-axis; (**b**) tracking error of Y-axis; (**c**) tracking error of yaw attitude.

**Figure 12:**
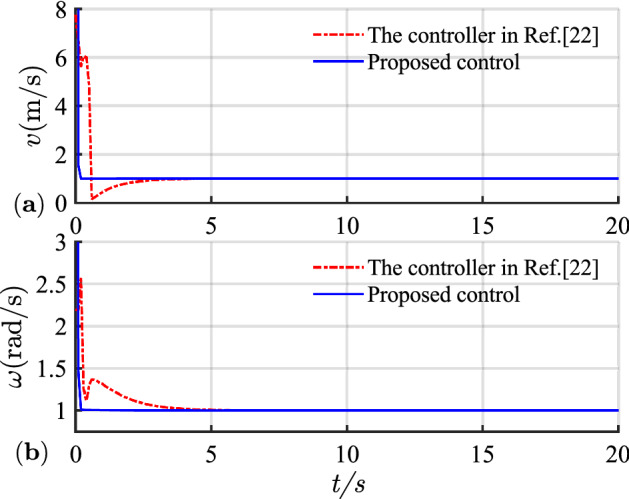
(**a**) Linear velocity; (**b**) angular velocity.

**Figure 13:**
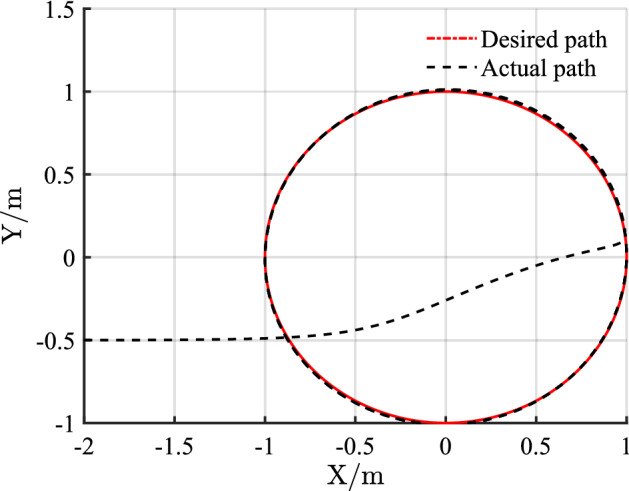
Circular trajectory tracking with disturbance.

**Figure 14:**
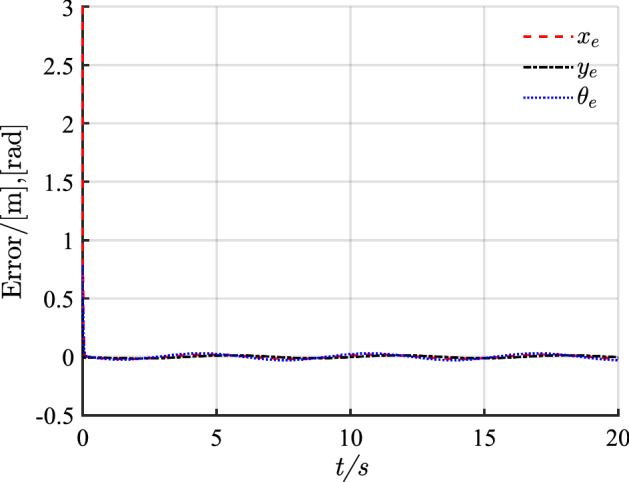
Pose error with disturbance.

**Figure 15:**
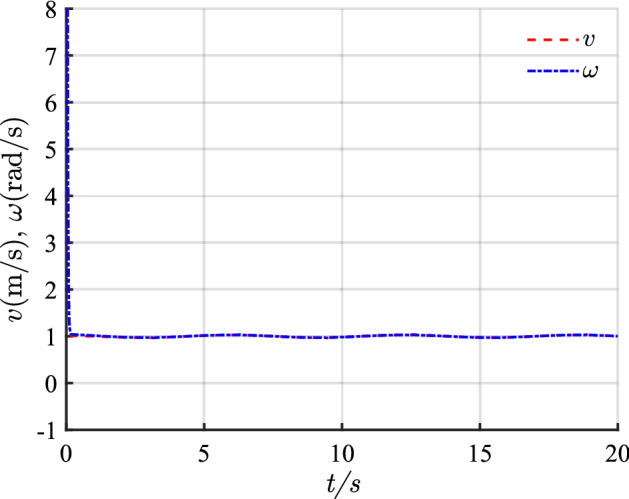
Control input with disturbance.

The simulation results of circular trajectory tracking for comparison with Ref.^22^ are shown in Figs. 10, 11 and 12, whereas the simulation results of circular trajectory tracking for comparison with the proposed control adding disturbance are shown in Figs. 13, 14 and 15.

Similarly, the proposed controller allows a fast and efficient tracking of the desired circular path with larger position and angle errors as shown in Fig. 10. Meanwhile, in Fig. 11, the X-axis, Y-axis, and yaw attitude tracking errors converge approximately 0.6, 6, and 6 earlier. The proposed controller also has a good tracking thought for circular paths. Simultaneously, the linear and angular velocities in the circular path simulation maintain their rapid convergence in Fig. 12.

Figure 13 shows that although the control system is subject to external periodic interference, the system can still maintain a good tracking accuracy after the system enters a steady state. Meanwhile, Fig. 14 shows that when disturbance is added, the pose error changes periodically, and the change range is small.

Combining Figs. 10, 11, 12, 13, 14 and 15 shows that the proposed controller not only ensures the accuracy and stability of AGV circular trajectory tracking but also overcomes the influence of external interference and demonstrates strong robustness.

The maximum positional error of the proposed controller after the system convergence is shown in Table 1.

**Table 1:** Maximum positional error.

Path	$x_{e\max } /{\text{m}}$	${\text{y}}_{{{\text{e}}\max }} /{\text{m}}$	$\theta_{{{\text{e}}\max }} /{\text{m}}$
linear path	1.1368E−04	9.2635E−04	9.2690E−04
circular path	1.1442E−04	9.6459E−04	9.6452E−04

As can be seen from the table, the proposed controller achieves path tracking well regardless of linear or circular paths. The maximum positional error is kept within the order of 10^–4^ after convergence, thereby suggesting that the proposed control system has good tracking accuracy.

## Conclusion

The proposed control approach addresses the tracking control problem of mobile AGV. Based on the improved reaching law, the proposed improved sliding mode controller achieves a higher convergence rate and tracking precision. Compared with the methods introduced in previous studies, the method proposed in this paper remains continuous and smooth by employing the hyperbolic tangent and continuous functions. As a result, the inherent chattering phenomenon in conventional sliding controls is eventually eliminated. The stability of the proposed control has been strictly demonstrated in mathematics, and the simulations manifest the effectiveness and superiority of the proposed rule.

## Data Availability

Data available on request from the corresponding author.
